# Size Tuning of Mesoporous Silica Adjuvant for One-Shot Vaccination with Long-Term Anti-Tumor Effect

**DOI:** 10.3390/pharmaceutics16040516

**Published:** 2024-04-08

**Authors:** Xiupeng Wang, Yu Sogo, Xia Li

**Affiliations:** Health and Medical Research Institute, Department of Life Science and Biotechnology, National Institute of Advanced Industrial Science and Technology (AIST), Central 6, 1-1-1 Higashi, Tsukuba 305-8566, Ibaraki, Japan; yu-sogou@aist.go.jp (Y.S.); li.xia@nims.go.jp (X.L.)

**Keywords:** adjuvant, cancer, particle size, one-shot vaccination

## Abstract

Despite recent clinical successes in cancer immunotherapy, it remains difficult to initiate a long-term anti-tumor effect. Therefore, repeated administrations of immune-activating agents are generally required in most cases. Herein, we propose an adjuvant particle size tuning strategy to initiate a long-term anti-tumor effect by one-shot vaccination. This strategy is based on the size-dependent immunostimulation mechanism of mesoporous silica particles. Hollow mesoporous silica (HMS) nanoparticles enhance the antigen uptake with dendritic cells around the immunization site in vivo. In contrast, hierarchically porous silica (HPS) microparticles prolong cancer antigen retention and release in vivo. The size tuning of the mesoporous silica adjuvant prepared by combining both nanoparticles and microparticles demonstrates the immunological properties of both components and has a long-term anti-tumor effect after one-shot vaccination. One-shot vaccination with HMS-HPS-ovalbumin (OVA)-Poly IC (PIC, a TLR3 agonist) increases CD4^+^ T cell, CD8^+^ T cell, and CD86^+^ cell populations in draining lymph nodes even 4 months after vaccination, as well as effector memory CD8^+^ T cell and tumor-specific tetramer^+^CD8^+^ T cell populations in splenocytes. The increases in the numbers of effector memory CD8^+^ T cells and tumor-specific tetramer^+^CD8^+^ T cells indicate that the one-shot vaccination with HMS-HPS-OVA-PIC achieved the longest survival time after a challenge with E.G7-OVA cells among all groups. The size tuning of the mesoporous silica adjuvant shows promise for one-shot vaccination that mimics multiple clinical vaccinations in future cancer immunoadjuvant development. This study may have important implications in the long-term vaccine design of one-shot vaccinations.

## 1. Introduction

Cancer immunotherapy is currently emerging as an important cancer treatment option. Different from conventional treatments of cancer, such as surgery, chemotherapy, and radiotherapy, which directly remove/kill cancer cells and surrounding healthy cells, cancer immunotherapy shows promise in utilizing the body’s own immune system to remove cancer cells without damaging surrounding healthy cells. Despite recent clinical successes in cancer immunotherapies, including immune checkpoint blockades, chimeric antigen receptor-T cells, and cancer vaccines, it remains difficult to achieve long-term therapeutic efficacy [1,2,3,4]. Furthermore, repeated administrations over months to years are necessary, imposing significant physical, psychological, and economic burdens on patients. Additionally, there are risks of treatment-associated toxicity, chronic administration pain, and poor adherence [5,6,7].

A too-short immunomodulator retention period around the vaccination site is one of the main obstacles to realizing long-term therapeutic responses, as a too-rapid clearance of immunomodulators is considered to decrease the quality and duration of generated immune memory [8,9]. Thus, a prolonged release of an immunomodulator is advantageous in not only generating stronger and persistent immune responses but also minimizing systemic side effects [6,10,11,12]. Previous studies showed that the time of immunomodulator exposure during vaccination is crucial to the initiation of long-term T cell memory [6,10,11,12]. For instance, sustained antigen exposure over several days induced the generation of many memory CD8^+^ T cells, whereas short-term antigen exposure induced a limited number of memory CD8^+^ T cells [6]. In addition, a prolonged antigen release from particulate vaccines resulted in enhanced antigen presentation, an increased number of cytotoxic CD8^+^ T cells, and thus, increased anti-tumor immunity in vivo [10]. The efficient activation of CD8^+^ T cells is highly affected by dendritic cells (DCs). Therefore, promoting DC recruitment and antigen delivery to DCs is essential for the activation of CD8^+^ T cells. In more detail, DCs are activated and become mature after the uptake of vaccine components. Then, the activated and mature DCs migrate to lymphoid organs, present antigens through the peptide/major histocompatibility complex (MHC) to naive T cells, and induce innate and adaptive immune responses. Such processes induce effector and memory cell proliferation and differentiation [13,14].

The size of the vaccine carrier adjuvants is a crucial determinant of the antigen retention period, antigen uptake, DC activation, and antigen presentation [15,16,17,18,19,20,21,22,23]. The efficiency of antigen presentation is determined by the antigen uptake pathway, which is, in turn, determined by particle size. Particles of 20–200 nm size are engulfed by endo- or pinocytosis, whereas particles bigger than 0.5 μm in size need phagocytosis [24,25]. Another study also showed that the uptake of mesoporous silica (MS) by HeLa cells is size-dependent. MS particles of 50 nm in size showed the maximum uptake by HeLa cells, compared with those 30, 110, 170, and 280 nm in size [21]. In addition, particle size is linked to antigen presentation, cytokine secretion, and the type of induced immune response [15,16,20,23]. Recently, we illustrated the size-dependent immunogenicity of hydroxyapatite (HA) rods. HA rods with lengths of 100, 200, and 500 nm promoted T-cell immunity by enhancing the uptake of the antigen by DCs, DC maturation, and antigen delivery to lymph nodes. On the other hand, HA rods with lengths of 500 nm–10 μm prolonged antigen retention and DC accumulation at injection sites, possibly owing to the low clearance rate of microparticles in vivo [22]. MS particles act both as immune enhancers and as delivery systems for biomolecules. Previous studies showed that MS improves Th1 and Th2 immunity and increases effector memory T cells in mouse models [4]. Therefore, this study focuses on the use of MS of different sizes to control the delivery of cancer antigens. In this study, we hypothesized that the size tuning of an adjuvant prepared by combining microparticles and nanoparticles can realize the long antigen retention of microparticles and the high antigen delivery efficiency of nanoparticles.

## 2. Materials and Methods

### 2.1. Synthesis of Template Carbonaceous Microspheres

Glucose (FUJIFILM Wako Pure Chemical Corporation, Osaka, Japan) was dissolved in ultrapure water (0.2 M) and added into a Teflon bottle (80 mL) held in a stainless autoclave at 180 °C for 4 h. Carbonaceous microspheres were collected after centrifugation (3000× *g*, 20 min). Then, they were washed in water or ethanol 5 times. The obtained product was dried at 80 °C for 4 h.

### 2.2. Synthesis of Hierarchically Porous Silica (HPS) Microparticles and Hollow Mesoporous Silica (HMS) Nanoparticles

HPS microparticles were synthesized using a tri-templating method. Typically, 1,3,5-trimethylbenzene (TMB, Tokyo Chemical Industry Co., Ltd., Tokyo, Japan), triblock copolymers EO106PO70EO106 (Pluronic F127, AnaSpec Inc., Fremont, CA, USA), and KCl (FUJIFILM Wako Pure Chemical Corporation, Osaka, Japan) were dissolved in 2 M HCl (FUJIFILM Wako Pure Chemical Corporation, Osaka, Japan) with stirring at 30 °C. Carbonaceous microspheres (1 wt%) were fully dispersed in TMB and F127 solution with ultrasonication. Then, tetraethoxysilane (TEOS, FUJIFILM Wako Pure Chemical Corporation, Osaka, Japan) was added dropwise with stirring at 600 rpm for 3 min. The molar ratio of TEOS:F127:TMB:H_2_O:KCl:HCl was 1:0.0037:0.5:155:3.36:6. Stirring was continued for 2 h at 30 °C. The mixture was placed in a Teflon bottle held in a stainless autoclave at 100 °C for 24 h. The precipitate was collected by centrifugation (3000× *g*, 20 min), washed with ultrapure water/ethanol, dried at 80 °C, and heat-treated at 550 °C for 5 h. HMS nanoparticles were synthesized using a Stöber solution composed of hexadecyltrimethylammonium bromide (CTAB, FUJIFILM Wako Pure Chemical Corporation, Osaka, Japan), TEOS, H_2_O, ammonia (FUJIFILM Wako Pure Chemical Corporation, Osaka, Japan), and ethanol (Wako) as described previously [4]. TEOS was added to solution containing CTAB, NH_3_, H_2_O, and C_2_H_5_OH with TEOS: CTAB: NH_3_: H_2_O: C_2_H_5_OH molar ratio of 1: 0.0922: 2.96: 621: 115 under stirring at 35 °C. After 24 h, the precipitate was collected by centrifugation (3000× *g*, 10 min). After washing with ethanol, the precipitate was mixed with ultrapure water at 70 °C for 2 h, collected by centrifugation (3000× *g*, 10 min), washed with ultrapure water/ethanol, dried at 80 °C, and heat-treated at 550 °C for 5 h to obtain the HMS nanoparticles.

### 2.3. Characterization of HPS Microparticles and HMS Nanoparticles

The morphology of the HPS microparticles and HMS nanoparticles was observed using a transmission electron microscope (TEM, EM-002B, TOPCON, Tokyo, Japan) and a field emission scanning electron microscope (FE-SEM, S-4800, Hitachi, Japan). In addition, the samples were analyzed using a powder X-ray diffractometer (XRD, Rigaku, Tokyo, Japan) employing CuKα X-rays. Fourier transform infrared (FTIR) spectra of the samples were recorded using an FTIR-350 spectrometer (JASCO Corporation, Tokyo, Japan). The zeta potential of the particles was analyzed using a Delta Nano C particle analyzer (Beckman Coulter, Inc., Brea, CA, USA). The nitrogen gas adsorption–desorption isotherm of HPS microparticles and HMS nanoparticles was measured using a specific surface area/pore size distribution analyzer (Micromeritics, Norcross, GA, USA).

### 2.4. In Vivo Cellular Uptake of Cancer Antigen

Fluorescein conjugates of ovalbumin (F-OVA, Life Technologies, Carlsbad, CA, USA) were used as the cancer antigen to test its in vivo cellular uptake. First, F-OVA (100 μg) was simply mixed with HMS nanoparticles or HPS microparticles (0.9 mg/100 μL) in saline and subcutaneously injected into the flank of a mouse (C57BL/6J, female, 6 weeks old, CLEA Inc., Tokyo, Japan). The mice were divided into four groups: (1) F-OVA, (2) HMS-F-OVA, (3) HPS-F-OVA, and (4) HMS-HPS-F-OVA. Cells around the injection site were collected on d3 to prepare a single-cell suspension. Non-specific staining was inhibited by anti-CD16/CD32 antibody (2.4G2, BD Pharmingen, San Jose, CA, USA). Then, the cells were stained using anti-mouse CD11c and anti-mouse CD86 antibodies (Biolegend, San Diego, CA, USA) for 30 min. Flow cytometry was performed using FACSAria (BD Bioscience, Franklin Lakes, NJ, USA).

### 2.5. In Vivo OVA Release Test

Alexa Fluor 647 conjugates of OVA (A647-OVA, Molecular Probe, Eugene, OR, USA) were used as the cancer antigen to test its in vivo release. A647-OVA (100 μg) was simply mixed with HMS nanoparticles or HPS microparticles (0.9 mg/100 μL) in saline, and the suspension was subcutaneously injected into the right flank of a mouse. The mice were divided into four groups: (1) A647-OVA, (2) HMS-A647-OVA, (3) HPS-A647-OVA, and (4) HMS-HPS-A647-OVA. A647-OVA remaining at the injection site was observed using an in vivo imaging system (IVIS, PerkinElmer, Shelton, CT, USA) between d0 and d3.

### 2.6. In Vivo Anti-Tumor Test

Chicken egg OVA (Sigma-Aldrich, St. Louis, MO, USA) was used as the cancer antigen-specific to E.G7-OVA lymphoma cells (CRL-2113™, ATCC, Manassas, VA, USA). First, OVA (100 μg) and Poly IC (PIC, 12.5 μg) were simply mixed with HMS nanoparticles or HPS microparticles (0.9 mg/100 μL) in saline and subcutaneously injected into the left flank of a mouse to elicit immune responses against OVA. The mice were divided into four groups: (1) OVA-PIC, (2) HMS-OVA-PIC, (3) HPS-OVA-PIC, and (4) HMS-HPS-OVA-PIC. Next, OVA (100 μg/100 μL in saline) was subcutaneously injected into the left flank of a mouse on d120 to induce the recall of immune responses against OVA. Then, E.G7-OVA cells (5 × 10^5^ cells/mouse) were subcutaneously injected into the right flank of a mouse on d124. The tumor size of a mouse was measured using a caliper. The mice showing no formation of a tumor larger than 15 mm were considered survivors. Flow cytometry analysis was conducted to study the mechanisms of anti-tumor immunity. Lymphocytes from the draining lymph nodes were obtained from mice 1 month after E.G7-OVA injection to prepare a single-cell suspension. The cells were blocked with an anti-CD16/CD32 antibody and stained with anti-mouse CD4, anti-mouse CD8α, and anti-mouse CD86 antibodies (Biolegend) for 30 min.

To further analyze the mechanisms of anti-tumor immunity provided by the different silica particles, OVA (200 μg) and PIC (50 μg) were simply mixed with HMS nanoparticles or HPS microparticles (2 mg/100 μL) in saline and subcutaneously injected into the left flank of a mouse to elicit immune responses against OVA. The mice were divided into five groups: (1) saline, (2) OVA-PIC, (3) HMS-OVA-PIC, (4) HPS-OVA-PIC, and (5) HMS-HPS-OVA-PIC. Next, OVA (100 μg/100 μL in saline) was subcutaneously injected into the left flank of a mouse on d104 to induce the recall of immune responses against OVA. Then, splenocytes were collected on d109 to prepare a single-cell suspension. The cells were blocked with an anti-CD16/CD32 antibody and stained with anti-mouse CD8a, anti-mouse CD44, anti-mouse CD62L (Biolegend), and anti-mouse T-Select H-2Kb OVA Tetramer-SIINFEKL (MBL) antibodies for 30 min. Moreover, the nearby draining lymph nodes were collected on d109, and microarray analysis was performed using Agilent SurePrint G3 Mouse GE Microarray 8 × 60 K Ver. 2.0. The data were analyzed by Metascape [26]. In addition, to confirm the safety of the suspension, major organs (heart, spleen, liver, kidney, and lung) from group (1) saline and group (5) HMS-HPS-OVA-PIC were collected for hematoxylin and eosin (HE) staining on d104.

### 2.7. Statistical Analysis

The statistical significance of differences was calculated by Student’s *t* test, ANOVA with Tukey’s multiple comparisons post hoc test, or log-rank test. A *p* value of <0.05 was considered statistically significant.

## 3. Results and Discussion

**Figure 1:**
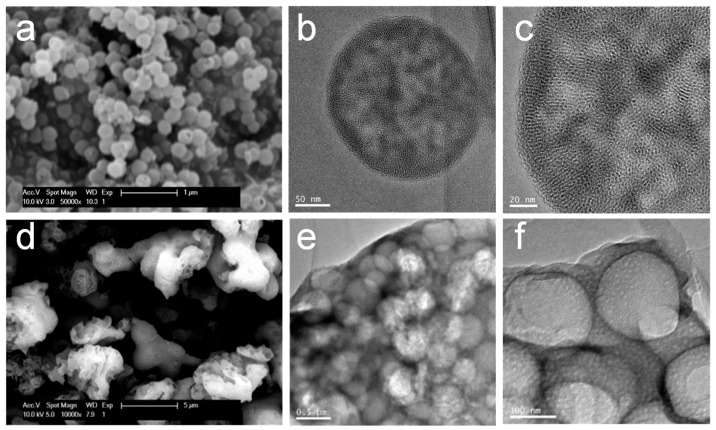
SEM (**a**,**d**) and TEM (**b**,**c**,**e**,**f**) images of HMS nanoparticles (**a**–**c**) and HPS microparticles (**d**–**f**).

**Figure 2:**
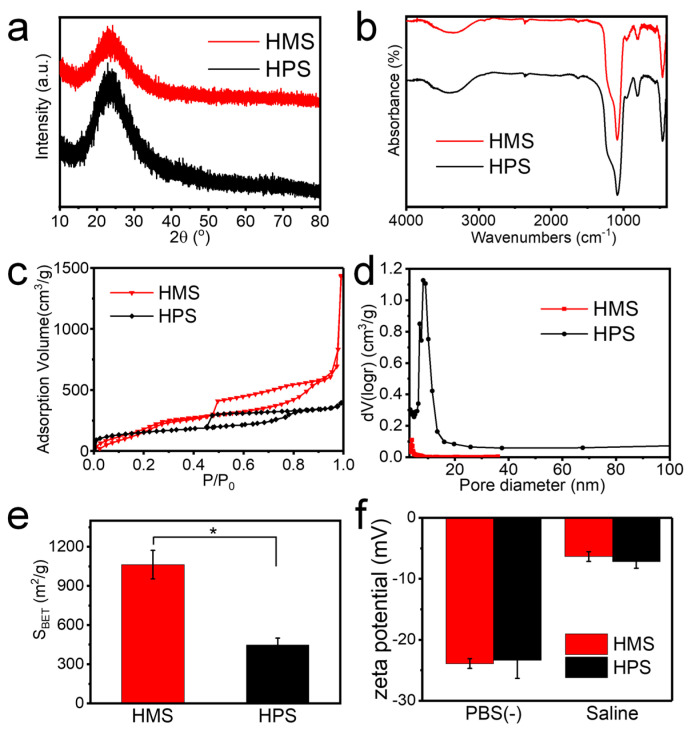
XRD patterns (**a**), FTIR spectra (**b**), N_2_ adsorption–desorption isotherms (**c**), pore size distribution curves (**d**), BET surface area (**e**), and zeta potential (**f**) of HMS nanoparticles and HPS microparticles (n = 3, * p < 0.05).

The HMS nanoparticles are about 200 nm in size with a hollow structure, and mesopores are about 2–6 nm in size on the shells (Figure 1a–c). The HPS microparticles are about 3–6 μm in size with hierarchical pores about 3–20 nm and 100 nm in size (Figure 1d–f). Both the HMS nanoparticles and the HPS microparticles are composed of amorphous silica, as shown by the XRD patterns and FTIR spectra (Figure 2a,b). The BET surface areas of the HMS nanoparticles and HPS microparticles are 1063 ± 109 and 448 ± 52 m^2^/g, respectively (Figure 2c–e). The HMS nanoparticles and HPS microparticles show zeta potentials of −6 and −7 mV in saline (pH around 5.5) and −24 and −23 mV in PBS(−) (pH around 7.4), respectively (Figure 2f). Both HMS nanoparticles and HPS microparticles are composed of amorphous silica and exhibited porous structures, high surface areas, and negative zeta potentials, so their different sizes may be one of the most important factors determining their immune response. Moreover, MS particles showed a high affinity to biomolecules. The interactions between MS particles and biomolecules include hydrophobic interactions, π − π stacking, electrostatic interactions, and hydrogen bonds. In a previous report, after simply mixing tumor antigens with HMS nanoparticles, up to 68% of the tumor antigens could be adsorbed on HMS nanoparticles. Furthermore, HMS nanoparticles prolonged the release of biomolecules, with only 33% of adsorbed tumor antigens released in saline after 7 days [4]. The HMS nanoparticles and HPS microparticles should be good carriers for antigens and immunostimulatory molecules, similar to previously reported MS particles, owing to their abundant mesopores, their high BET surface area, and the Si-OH groups on MS particles [27,28,29].

**Figure 3:**
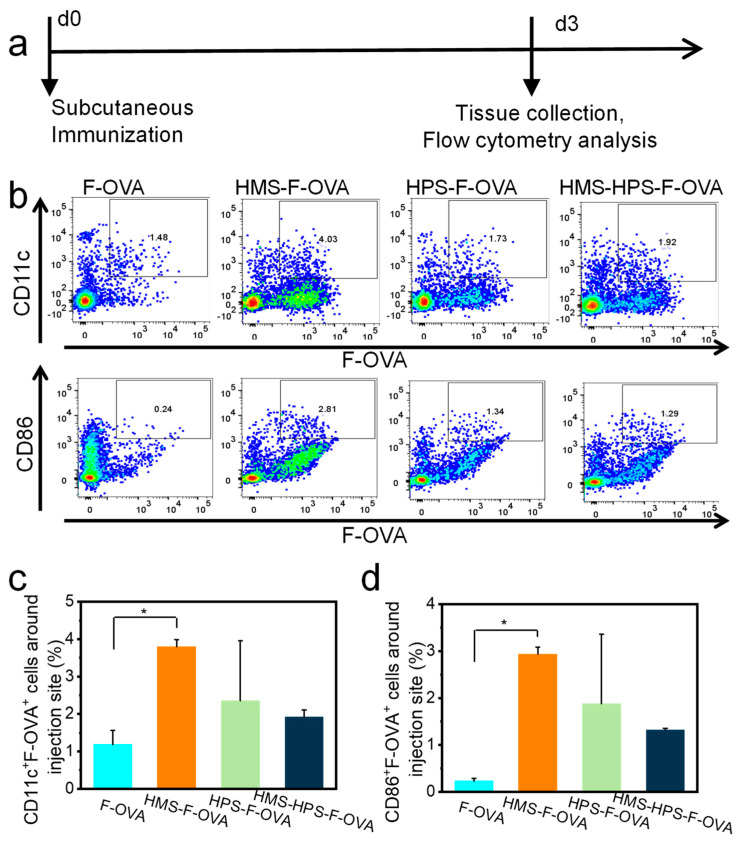
HMS nanoparticles promoted cellular uptake of F-OVA around injection site in vivo. Experimental protocol (**a**); CD11c^+^F-OVA^+^ cells around injection site (**b**,**c**); CD86^+^F-OVA^+^ cells around injection site (**b**,**d**) 3 days after immunization (n = 3, * p < 0.05).

**Figure 4:**
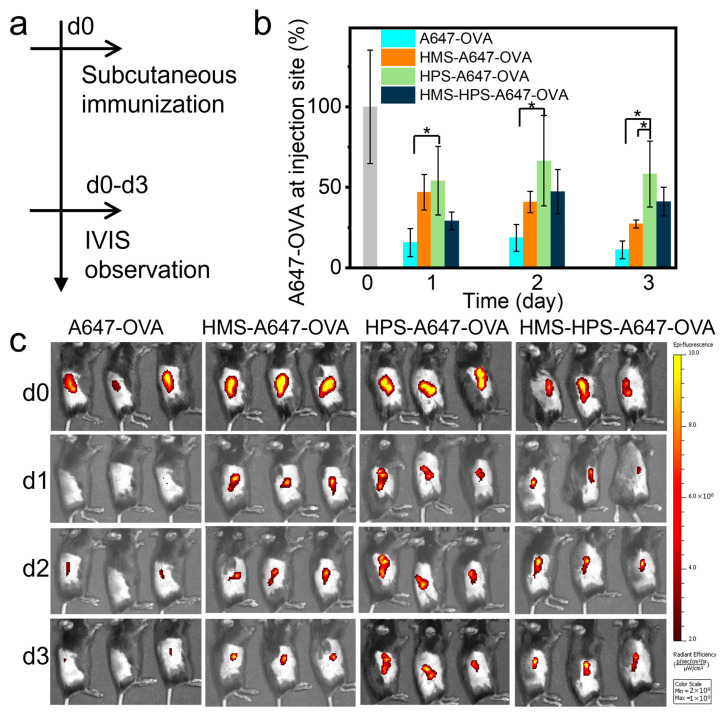
HPS microparticles prolonged A647-OVA retention time around injection site in vivo. Experimental protocol (**a**); percentage of A647-OVA remaining at the injection site observed by an in vivo imaging system (**b**,**c**) (n = 3, * *p* < 0.05). The data in d0 of b were calculated from A647-OVA, HMS-A647-OVA, HPS-A647-OVA, and HMS-HPS-A647-OVA.

Firstly, the HMS nanoparticles greatly increase the antigen uptake by DCs around the injection site (Figure 3). Cells around the injection site were analyzed by flow cytometry 3 days after subcutaneous immunization. Mouse DC markers, including CD11c and CD86, are used to identify DCs in this study. Mice subcutaneously injected with F-OVA, HMS-F-OVA, HPS-F-OVA, and HMS-HPS-F-OVA show CD11c^+^F-OVA^+^ populations of 1.2%, 3.8%, 2.4%, and 1.9% around the injection site in vivo, respectively. In addition, mice subcutaneously injected with F-OVA, HMS-F-OVA, HPS-F-OVA, and HMS-HPS-F-OVA show CD86^+^F-OVA^+^ populations of 0.2%, 2.9%, 1.9%, and 1.3% around the injection site in vivo, respectively. Mice subcutaneously injected with HMS-F-OVA show the highest percentages of CD11c^+^F-OVA^+^ and CD86^+^F-OVA^+^ cells around the injection site among the four subcutaneously injected groups in this study (Figure 3). Previous studies showed that the delivery of F-OVA can be easily degraded under enzymatic or physiological conditions in vivo [30], which explains why solely delivered F-OVA is not present at the injection site (Figure 4) and is not phagocytosed by DCs (Figure 3).

Nano-sized particles tend to be engulfed by DCs; thus, antigens and immunomodulatory molecules loaded in nano-sized particles can also be efficiently internalized in DCs [22]. Efficient vaccine component delivery to DCs is an essential step for the initiation of adaptive immune responses. This is because vaccine component delivery to DCs promotes DC activation and maturation, enhances MHC class II presentation and MHC class I cross-presentation to naïve T cells, and promotes the initiation of adaptive immune responses [30]. Previous studies showed that MS nanoparticles with a diameter of about 100 nm acted as good carriers to internalize the cancer antigen in DCs; promoted the secretion of antigen-specific interleukin-2 (IL-2), IL-4, IL-10, interferon-γ (IFN-γ), immunoglobulin G (IgG), IgG1, and IgG2a; increased the number of CD4^+^ and CD8^+^ T cells and effector memory CD4^+^ and CD8^+^ T cells; and consequently, enhanced anti-tumor immunity in vivo [4,31].

Secondly, the HPS microparticles prolong cancer antigen retention in vivo (Figure 4). A647-OVA remaining at the injection site was tested using an in vivo imaging system on d0–d3. For mice subcutaneously injected with A647-OVA, the fluorescence signal intensity decreases very rapidly 1 day after injection. However, for mice injected with HMS-A647-OVA, HPS-A647-OVA, and HMS-HPS-A647-OVA, the fluorescent signal intensity decreased very slowly 1–3 days after injection. Mice injected with HPS-A647-OVA showed the highest fluorescence signal intensity among all groups 3 days after injection (Figure 4). The percentages of A647-OVA remaining around the injection site for mice injected with A647-OVA, HMS-A647-OVA, HPS-A647-OVA, and HMS-HPS-A647-OVA 3 days after subcutaneous injection are 11.2%, 27.3%, 58.3%, and 41.2%, respectively (Figure 4). Mice injected with HMS-A647-OVA and HMS-HPS-A647-OVA show lower fluorescence signal intensity 3 days after injection than mice injected with HPS-A647-OVA (Figure 4). This is because HMS-A647-OVA with an HMS size of 200 nm (Figure 1a–c) can be phagocytosed efficiently by immune cells (e.g., DCs) and delivered to other parts of the body (Figure 1d–f). These results are consistent with previously reported size-dependent immunogenicity of particulate adjuvants [22]. Moreover, the effect of antigen release kinetics on immune responses was shown in previous studies [3,6,32,33]. The prolonged retention of the antigen in the injection site will enhance the recruitment and activation of APCs, which is important for priming long-term CD8 T-cell immunity [32,33].

**Figure 5:**
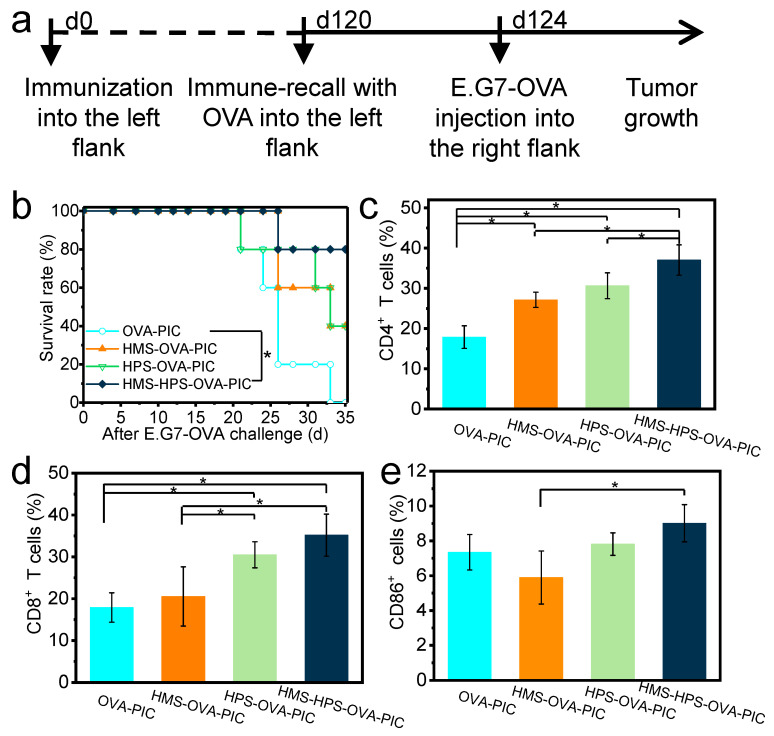
HMS nanoparticles and HPS microparticles showed synergistic anti–tumor immunity in vivo. Experimental protocol (**a**), survival rate of mice (**b**) (n = 5, * *p* < 0.05). HMS-HPS-OVA-PIC immunized mice showed highest CD4^+^, CD8^+^, and CD86^+^ (quantitative results, (**c**–**e**)) cells in draining lymph nodes of mice (n = 5, * *p* < 0.05).

Lastly, the combination of HMS nanoparticles and HPS microparticles is found to exhibit the immunological capabilities of both components and achieve long-term anti-tumor immune responses in vivo (Figure 5). We then evaluated the longevity and efficiency of the memory recall response in mice immunized with HMS-HPS-OVA-PIC, HMS-OVA-PIC, HPS-OVA-PIC, and OVA-PIC after immunization with OVA on d120. Then, the mice were challenged with E.G7-OVA cells on d124. Mice challenged with E.G7-OVA cells 124 days after a single injection of HMS-HPS-OVA-PIC show the highest survival rate (80%) among those immunized with HMS-OVA-PIC, HPS-OVA-PIC, and OVA-PIC, which show survival rates of 40%, 40%, and 0% at the endpoint, respectively (Figure 5a,b). Mice immunized with OVA-PIC, HMS-OVA-PIC, HPS-OVA-PIC, and HMS-HPS-OVA-PIC show CD4^+^ T cells of 17.9%, 27.2%, 30.7%, and 37.1%; CD8^+^ T cells of 17.9%, 20.5%, 30.5%, and 35.2%; and CD86^+^ cells of 7.3%, 5.9%, 7.8%, and 9.0% in draining lymph nodes of mice at the endpoint, respectively. The data indicate that the one-shot vaccination with HMS-HPS-OVA-PIC can definitely increase the populations of CD4^+^ T cells, CD8^+^ T cells, as well as CD86^+^ cells in the draining lymph nodes (Figure S1). The activation of CD4^+^ T cells, CD8^+^ T cells, and DCs is essential for inducing anti-tumor immune responses [34]. Poly IC, a synthetic analog of dsRNA and an agonist of Toll-like receptor 3 (TLR3), was used to stimulate anti-tumor immunity as an adjuvant [35]. TLR3 is widely expressed in neurocytes, fibroblasts, immune cells, and epithelial cells [36]. Poly IC induces Th1-based anti-cancer immunity, stimulates DC activation, increases the proliferation of antigen-specific CD4^+^ and CD8^+^ T cells, and promotes the secretion level of cytokines [37].

**Figure 6:**
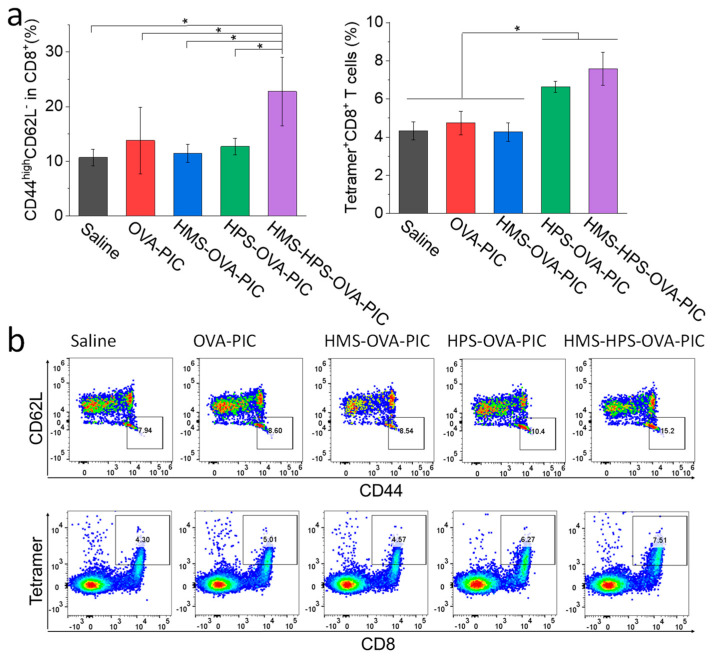
Quantitative (**a**) and representative (**b**) results of effector memory CD44^high^CD62L^–^ in CD8^+^ T cells and tumor–specific tetramer^+^CD8^+^ T cells in splenocytes of mice (n = 3, * *p* < 0.05).

The anti-tumor mechanism induced by one-shot vaccination with HMS-HPS-OVA-PIC was further studied using splenocytes collected from the vaccinated mice. One-shot vaccination with saline (as negative control), OVA-PIC, HMS-OVA-PIC, HPS-OVA-PIC, and HMS-HPS-OVA-PIC, respectively, show the following percentages of cells: effector memory CD44^high^CD62L^−^ cells among CD8^+^ T cells, 10.7%, 13.8%, 11.5%, 12.7%, and 22.8%; tumor-specific tetramer^+^CD8^+^ T cells among splenocytes, 4.3%, 4.7%, 4.3%, 6.6%, and 7.6%. The data indicate that the one-shot vaccination with HMS-HPS-OVA-PIC can definitely increase the populations of effector memory CD44^high^CD62L^−^ cells among CD8^+^ T cells and tumor-specific tetramer^+^CD8^+^ T cells among splenocytes 109 d after vaccination (Figure 6). Previous studies show that sustained and effective immune modulator delivery is critical for T cell activation and memory T cell initiation [6,10,11,12]. The HMS nanoparticles promote antigen cellular uptake (Figure 3) but do not significantly prolong antigen retention time (Figure 4). On the contrary, HPS microparticles prolong antigen retention time (Figure 4) but do not significantly promote antigen cellular uptake (Figure 3). These results also show that antigen retention time and cellular uptake by immune cells are both essential for activating an effective and long-lasting immune response. These results are consistent with previous publications [6,10,11,12].

**Figure 7:**
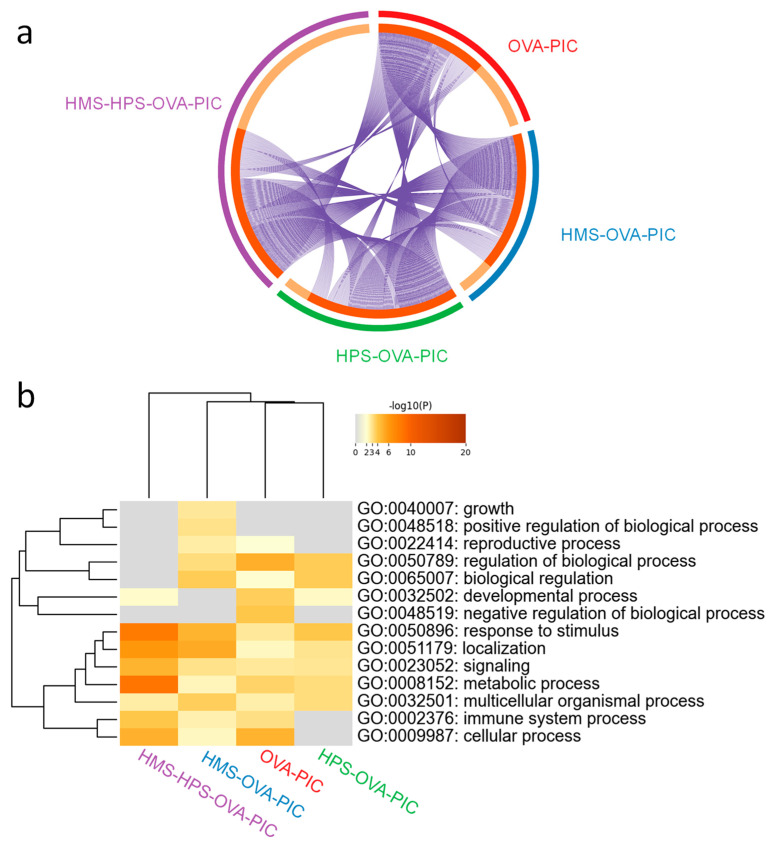
Gene overlap analysis (**a**); Heatmap of the top–level gene ontology biological processes, colored by *p*-values (**b**).

**Figure 8:**
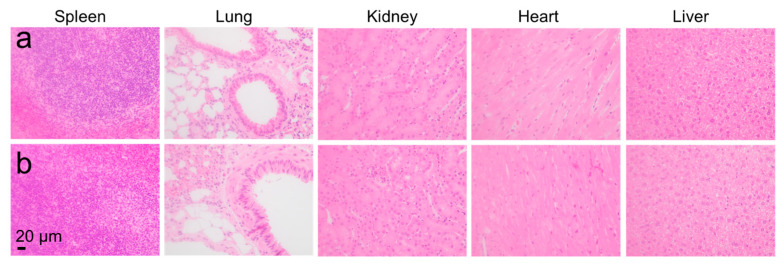
Representative histological pictures of major organs stained with H&E at d104 after one–shot vaccination. Saline (**a**); HMS-HPS-OVA-PIC (**b**).

To support the underlying anti-tumor mechanism of the one-shot vaccination, nearby draining lymph nodes were collected on d109 and investigated by microarray analysis. Microarray analysis results show that one-shot vaccination with HMS-HPS-OVA-PIC shows the highest number of shared genes (dark orange color in Figure 7a) and unique genes (light orange color in Figure 7a) among all groups. In contrast, one-shot vaccination with OVA-PIC shows the lowest number of shared genes (dark orange color in Figure 7a) among all groups. Moreover, one-shot vaccination with HMS-HPS-OVA-PIC shows markedly increased top-level gene ontology biological processes, including response to stimulus, localization, signaling, the metabolic process, the immune system process, and the cellular process (Figure 7b). The microarray results are consistent with the anti-tumor results (Figure 5b) and immune activation results (Figure 5, Figure 6, and Figure S1). Moreover, representative HE-stained sections of the spleen, lung, kidney, heart, and liver of mice one-shot-vaccinated with HMS-HPS-OVA-PIC indicate no marked histological changes compared with those of mice vaccinated with saline (Figure 8). The results suggest that HMS-HPS-OVA-PIC at an appropriate amount can be safely injected subcutaneously with a one-shot vaccination.

**Scheme 1:**
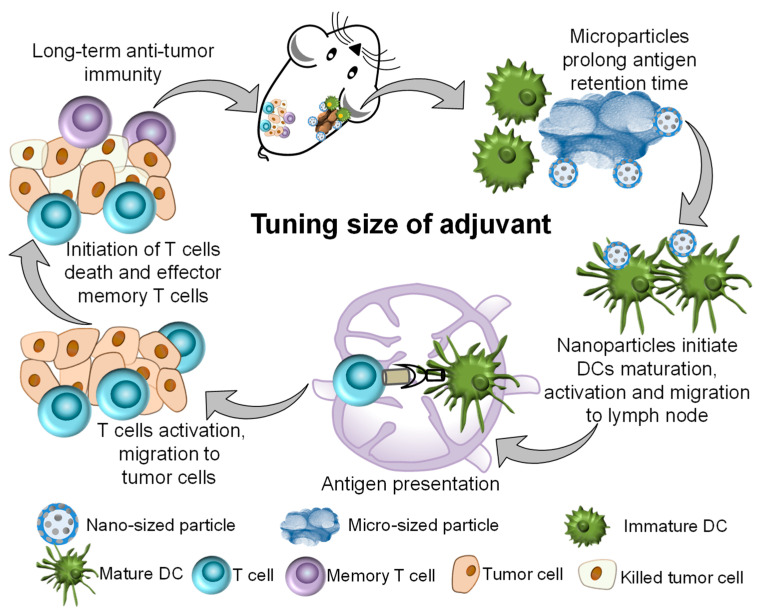
Size tuning of mesoporous silica adjuvant for one-shot vaccination with long–term anti–tumor effect.

Herein, mice challenged with E.G7-OVA cells 124 d after the one-shot vaccination with HMS-HPS-OVA-PIC show the highest anti-tumor immunity and the highest populations of effector memory CD44^high^CD62L^–^ cells among CD8^+^ T cells and tumor-specific tetramer^+^CD8^+^ T cells (Figure 5, Figure 6, and Figure S1), indicating a memory effect of anti-tumor immune responses for at least 4 months in mice. Immunological memory was mainly found in T cells, B cells, etc. After the elimination of the pathogen/antigen, most effector cells die, but a small number of memory T cells, B cells remain to maintain long-term memory that affects the specific pathogen/antigen (Scheme 1). Immunological memory is one of the core characteristics of adaptive immune responses; it provides an enhanced and rapid immune response to a previously encountered pathogen/antigen [38]. Moreover, most present vaccination schedules require several administrations at set time intervals. In our previous cancer immunoadjuvant studies using preventative and re-challenge models, mice were generally vaccinated three to four times within 4–10 d, then challenged with tumor cells 4–19 d after the last vaccination [15,22,31,39]. Herein, one-shot vaccination with combined micro-sized and nano-sized particles initiated long-term anti-tumor immune responses since the adjuvant particle grading strategy combining micro-sized and nano-sized particles balanced both the antigen retention benefits of micro-sized particles and the significant antigen delivery benefits of nano-sized particles. Experiments directly comparing this regime with traditional multi-immunization regimens should be further studied.

## 4. Conclusions

The immune stimulation mechanisms of two types of mesoporous silica particles (HMS nanoparticles and HPS microparticles) were illustrated in this study. The HMS nanoparticles are about 200 nm in size with a hollow structure and mesopores of about 2–6 nm on the shells, whereas the HPS microparticles are about 3–6 μm in size with hierarchical pores about 3–20 nm and 100 nm in size. The HMS nanoparticles enhance antigen uptake by DCs around the immunization site in vivo. The HPS microparticles prolong cancer antigen retention and release in vivo. Mice challenged with E.G7-OVA cells 4 months after one-shot vaccination with HMS-HPS-OVA-PIC show the highest anti-tumor immunity and the highest CD4^+^, CD8^+^, and CD86^+^ cell populations in draining lymph nodes of mice at the endpoint compared with those immunized with HMS-OVA-PIC, HPS-OVA-PIC, and OVA-PIC. Moreover, mice with one-shot vaccination with HMS-HPS-OVA-PIC show the highest number of effector memory CD8^+^ T cells and tumor-specific tetramer^+^CD8^+^ T cells in splenocytes among all groups. Thus, the size tuning of the adjuvant prepared by combining both HMS nanoparticles and HPS microparticles shows the long-term anti-tumor effect of one-shot vaccination.

## Data Availability

The data presented in this study are available upon request from the corresponding author.

## Supplementary Materials

The following supporting information can be downloaded at: https://www.mdpi.com/article/10.3390/pharmaceutics16040516/s1, Figure S1. Representative results of CD4^+^ (a), CD8^+^ (b) and CD86^+^ (c) in draining lymph node of mice.
