# Supplementary material for: Size Tuning of Mesoporous Silica Adjuvant for One-Shot Vaccination with Long-Term Anti-Tumor Effect
Source: Pharmaceutics. 2024 Apr 8;16(4):516. doi: 10.3390/pharmaceutics16040516 (PMC11053635; doi:10.3390/pharmaceutics16040516)
Supplement: Supplementary file 1 [file pharmaceutics-16-00516-s001.zip › pharmaceutics-2917112-supplementary.pdf]

Supporting Information

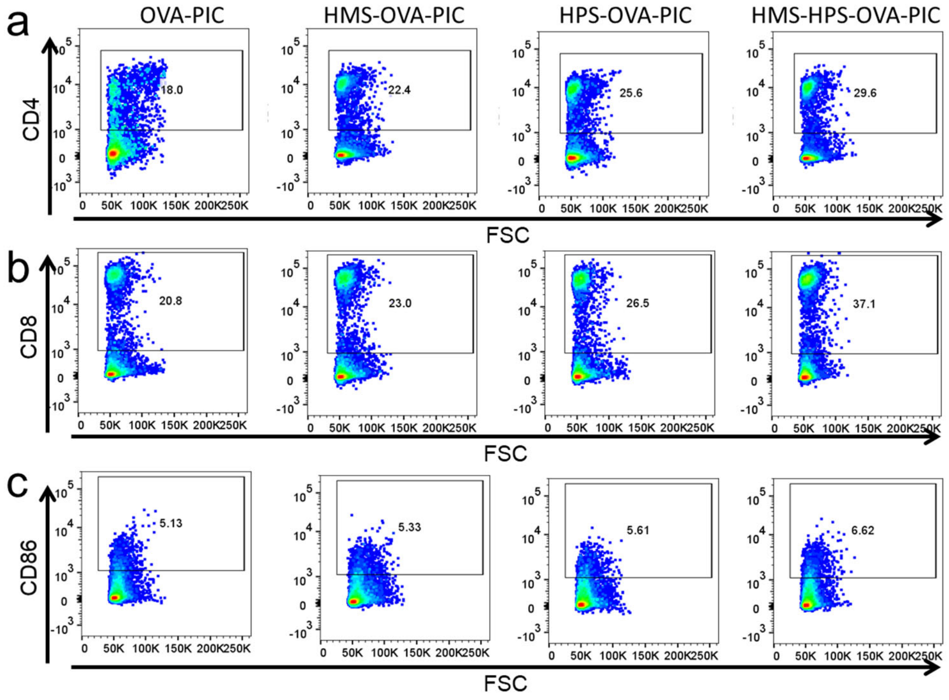

Figure S1. Representative results of CD4+ (a), CD8+ (b) and CD86+ (c) in draining lymph node of mice (n=5, $p<0.05$).
